# Analysis of antiretroviral therapy switch rate and switching pattern for people living with HIV from a national database in Japan

**DOI:** 10.1038/s41598-022-05816-5

**Published:** 2022-02-02

**Authors:** Toshio Naito, Hirotake Mori, Kazutoshi Fujibayashi, Shinichi Fukushima, Mayumi Yuda, Nobuyuki Fukui, Shotaro Tsukamoto, Mai Suzuki, Keiko Goto-Hirano, Ryohei Kuwatsuru

**Affiliations:** 1 Department of General Medicine, Juntendo University Faculty of Medicine, Hongo 2-1-1, Bunkyo-ku, Tokyo 113-8421 Japan; 2 Center for Promotion of Data Science, Juntendo University Graduate School of Medicine, Hongo 2-1-1, Bunkyo-ku, Tokyo 113-8421 Japan; 3 Department of Cardiovascular Biology and Medicine, Juntendo University Faculty of Medicine, Hongo 2-1-1, Bunkyo-ku, Tokyo 113-8421 Japan; 4 Department of Radiology, Juntendo University Faculty of Medicine, Hongo 2-1-1, Bunkyo-ku, Tokyo 113-8421 Japan

**Keywords:** Infectious diseases, HIV infections

## Abstract

To report the status of switch rates and time-to-switch of antiretroviral therapy (ART) regimens by evaluating anchor drug classes and common switching patterns in Japanese people living with human immunodeficiency virus (HIV, PLWH). This cross-sectional cohort study extracted data of 28,089 PLWH from the National Database of Health Insurance Claims and Specific Health Checkups of Japan (NDB), which contains data representing the entire population of Japan. PLWH with first prescription records of ART administered between January 2011 and March 2019 were identified (n = 16,069). The median time-to-switch and switch rates of anchor drug classes were estimated by Kaplan–Meier analysis. Brookmeyer–Crowley and Greenwood methods were used to estimate 95% confidence intervals for switch rates and median days, respectively. Switch rates were compared between anchor drug classes by year using log-rank tests. A total of 3108 (19.3%) PLWH switched anchor drug classes from first to second regimens. Switch rates increased continuously over 8 years for non-nucleoside reverse transcriptase inhibitors (NNRTIs) (14.9–65.5%) and protease inhibitors (PIs) (13.2–67.7%), with median time-to-switch of 1826 and 1583 days, respectively. Integrase strand transfer inhibitors (INSTIs) maintained a low switch rate (3.0–7.6%), precluding median-days calculation. Overall, the majority of patients treated initially with NNRTIs and PIs switched to INSTIs regardless of switching times (< 1 year: 67.3% and 85.9%, respectively; ≥ 1 year: 95.5% and 93.6%, respectively). The foremost switching strategies for first-to-second ART regimens are from NNRTIs or PIs to INSTIs regimens that maintain low switch rates long term. There was no observable difference in trend between sex, age and status of AIDS disease at first ART regimen. INSTIs HIV agents may be the most durable anchor drug class for PLWH receiving ART.

## Introduction

Antiretroviral drugs are being used globally to treat people living with human immunodeficiency virus (HIV, PLWH). International and national guidelines stipulate that durable, straightforward antiretroviral therapy (ART) regimens are the main focus of lifelong chronic HIV treatment^1–3^. Administration of ART regimen supports immune system function, reduces complications, and improves quality of life^4^, decreasing morbidity and mortality. Increased survival rates among PLWH are attributed to successful ART^5–7^. Life expectancy for PLWH now approximates that of HIV-negative individuals^8,9^.

Nevertheless, PLWH frequently switch ART regimens during chronic HIV treatment. Changes may occur because patients or clinicians think a new medication may produce better results or patients’ may become dissatisfied with their current regimen. Drug toxicity, unsatisfactory levels of viral suppression, drug-related adverse events (AEs), or just simplification of a regimen may also prompt a switch^10–12^. However, no consensus exists regarding switching strategies, and though changes occur frequently, switching strategies remain to be clearly defined.

The absence of a standard switching strategy makes it imperative to fully understand the circumstances most often leading to ART regimen switches and the anchor drug classes involved. Anchor drug classes that tend to be administered for longer durations also need to be identified.

Five classes of antiretroviral medications are used in Japan: nucleoside reverse transcriptase inhibitors (NRTIs), non-nucleoside reverse transcriptase inhibitors (NNRTIs), protease inhibitors (PIs), integrase strand transfer inhibitors (INSTIs), and entry inhibitors (EIs)^3^. The most recent guidelines for initiating an ART regimen recommend combination regimens (cART) consisting of two NRTIs as backbone therapy, with a third “anchor” drug from another class, most often NNRTIs, PIs, or INSTIs^1–3^. Clinicians’ selection of an anchor drug is central to the treatment strategy because backbone choices are comparatively limited.

Our previous study was a preliminary assessment of switch rates and time-to-switch of ART regimens using a hospital claims database with a distinctly smaller dataset of eligible HIV-positive patients^13^. We hypothesized that expanding the data source would not only confirm our preliminary results, but also update our knowledge of current switching rates and patterns among subgroups of patients with different comorbidities, sex and age. Therefore, we used the nationwide database, the National Database of Health Insurance Claims and Specific Health Checkups of Japan (NDB), which contains data representing the entire population in Japan, to confirm and update our preliminary findings and identify the most effective ART approaches for long-term treatment of PLWH in Japan.

## Methods

### Study design and data source

This observational, retrospective cohort study extracted patient data from the Japanese NDB for HIV-positive patients who received treatment between April 2009 and March 2019^14^. The NDB is the largest nationwide cross-sectional database in Japan, and it contains comprehensive health insurance claims records from the National Health Insurance system of Japan for direct primary care delivered as inpatient care. The NDB has been used to supported clinical studies^15,16^. The NDB includes data for diagnoses, age, sex, dates of outpatient services, dates of admission and discharge, procedures undertaken, diagnoses, drug prescriptions, and health checkup data. Patients treated from April 2009 to March 2019 with at least one diagnosis and any treatment processed in the claims data were enrolled. All included diagnoses were categorized according to the “The International Classification of Disease, 10th Revision, Clinical Modification” (ICD-10) diagnostic system. Patients with a diagnostic code of HIV-2 infection were excluded.

All insurance claims data are deidentified by the Ministry of Health, Labour and Welfare, and the ministry’s guidelines on information security were followed in the study. To ensure patient privacy, inspection by and permission from the Ministry for publication is required before submission of the draft manuscript.

### Study population

HIV-positive patients in the database were identified by the presence of at least one record of the International Classification of Diseases 10th Revision (ICD-10) codes B20‒24, including: HIV disease resulting in infectious and parasitic diseases (B20), malignant neoplasms (B21), other specified diseases (B22), other conditions (B23), and unspecified HIV disease (B24). To avoid including doubtful HIV-positive patients (i.e., poorly recorded or intentionally recorded for a claim), patients were required to have at least one prescription record of ART. The corresponding ICD-10 codes are listed in Supplemental Table 1. ART was defined as a prescription for any of the following antiretroviral drugs: NRTIs, NNRTIs, PIs, INSTIs, or EIs.

Data of patients meeting these criteria (n = 28,089) during the study period were extracted from the database. Patients who had received first prescriptions for an ART regimen between 2011 and 2019 (n = 16,069) were included in the present analysis.

### Outcomes

Primary outcomes were switch rates and time-to-switch associated with individual ART regimens by anchor drug classes. Secondary outcomes were switch rates and time-to-switch associated with anchor drug class-based ART regimens by type of backbone drug, characteristics of patients who experienced one anchor drug switch in each anchor drug class-based ART regimen, and common switching patterns of anchor drug classes.

### Definitions

Data extracted from the NDB included patients’ demographic characteristics (age, sex), and clinical characteristics, including year of first ART record in the database, prescription records of anchor drugs and backbone drugs, comorbidities, hospitalization history, and AIDS-defining illnesses. In this study, age was defined as the age of the patient at time of the first ART regimen.

#### Anchor drugs and backbone drugs

According to treatment guideline an ART regimen generally consists of two nucleoside reverse transcriptase inhibitors (NRTIs) administered in combination with a third active ART drug from one of three drug classes: INSTI, NNRTI, or PI with a pharmacokinetic enhancer (also known as a booster)^1,2^. In our study we referred to nucleoside reverse transcriptase inhibitors (NRTIs) as backbone drugs; and anchor drug is the third active ART drug.

The anchor drugs of the ART regimens were identified using receipt codes and were classified into three anchor drug classes according to the anti-HIV drug classification available in Japan^3^: (1) NNRTIs, (2) PIs, or (3) INSTIs.

The backbone drugs of the ART regimens were identified using receipt codes and classified into four categories: (1) tenofovir disoproxil fumarate (TDF); (2) abacavir (ABC); (3) tenofovir alafenamide fumarate (TAF); and (4) others.

#### ART regimen switch and time-to-switch

An ART regimen switch involved only anchor drug classes and was defined on the basis of a switch in the specific anchor drug class used in the ART regimen. The time-to-switch of an ART regimen was defined as the period from the date of the first record of anchor drug class in the ART regimen (defined as the first regimen) recorded within patient data (index date) to the date of switching to another anchor drug class in the subsequent ART regimen (defined as the second regimen) during the study period. The date of an anchor drug class switch was defined as the date of prescription of the new anchor drug class after the termination of the preceding (first) ART regimen. A regimen was considered discontinued when no initiation of any new anchor drug class was identified after termination of the preceding (first) ART regimen. A change of anchor drug within the same anchor drug class was not considered a switch. Patients with multiple prescription records of anchor drug class on the index date, patient who switched ART in the month following initial ART prescription and had more than one ART within the same month of the switch were also excluded.

#### AIDS-defining illnesses

AIDS-defining illnesses were identified by the presence of any of the following records prior to index date: HIV non-tuberculous mycobacteria, HIV cytomegalovirus infection, HIV candidiasis, HIV *Pneumocystis carinii* pneumonia, HIV Kaposi’s sarcoma, HIV Burkitt’s lymphoma, HIV non-Hodgkin’s lymphoma, HIV encephalopathy, HIV-associated dementia, slim disease, acquired immune deficiency syndrome, AIDS, neonatal HIV infection, and AIDS-related complex. The corresponding ICD-10 codes are listed in Supplemental Table 1.

Since there was no disease severity information in the NDB database, patients were sub-grouped according to status of AIDS diseases at the time of the first ART regimen. Patients with diagnosis of AIDS disease after initiation of 1st ART regimen were defined as no AIDS.

#### Comorbidities

Comorbidities were identified if any ICD-10-coded chronic illnesses were present prior to the index date, including: HIV-related diseases, hypertension, dyslipidemia, hepatitis B/C coinfection, diabetes mellitus, bone disorder, vascular disease, psychiatric disorders, kidney disease, and malignancy. Corresponding ICD-10 codes are listed in Supplemental Table 1.

#### History of hospitalization

A history of hospitalization was identified if a record of hospitalization was present before the ART regimen was prescribed.

### Statistical analysis

The proportions of anchor drug class-based and backbone-based drugs prescribed on the index date were obtained by year. Demographic and clinical characteristics of all patients on ART regimens were analyzed descriptively according to the anchor drug class prescribed on the index date. The median time-to-switch and switch rates according to anchor drug class prescribed on the index date and those stratified by the backbone drugs were estimated using Kaplan–Meier analysis. To estimate 95% confidence interval (CIs), the Brookmeyer and Crowley method was used for the median number of days, and the Greenwood method was used for switch rates. Log-rank tests were used to compare switch rates between the respective drug classes in each year. The Bonferroni method was performed to adjust p-values on multiple comparison. Discontinuation or continuation of the regimen to the end of the study period was censored.

The demographic and clinical characteristics of patients who switched anchor drug classes in their ART regimens were analyzed descriptively according to the anchor drug class prescribed on the index date. Timings of < 1 and ≥ 1 year were analyzed descriptively for patients who switched anchor drug classes in their ART regimens according to the anchor drug class prescribed on the index date and the corresponding 95% CI using Wilson scores.

Confounding factors for switching ART regimens and factors interacting with the anchor drug class were selected a priori based on previous studies. Time-to-switch was an objective variable and the anchor drug class, risk factors, and interaction term between anchor drug classes and each risk factor were included as explanatory variables in this model. The hazard ratio (HR) of each anchor drug class was calculated after adjusting for remaining variables and stratified by interaction factors to estimate the risk of switching anchor drug classes from the ART regimen prescribed on the index date. All statistical analyses were performed in the R 4.0.3 environment (R Core Team, 2020). All remaining statistical tests were two-sided, and *p* < 0.05 indicated statistical significance.

### Ethics statement

This study was performed in compliance with national regulations and institutional policies. The study protocol was approved by the Institutional Review Board (IRB) of Juntendo University Hospital (IRB #20-036). Because all patient data in the NDB were deidentified, informed consent was waived by the Institutional Review Board (IRB) of Juntendo University Hospital (IRB #20-036).

## Results

### Patient disposition

A total of 28,089 patients in the database had a coded diagnostic record of HIV during the study period. Of these, 27,912 were prescribed anchor drugs. After excluding 866 patients for whom multiple anchor drug classes were prescribed on the index date, who switched ART within the month following initial ART prescription, had more than one ART within the same month of the switch, or patients with prescription records of EIs as the first anchor drug, there were 27,046 patients who had a single prescription record of an anchor drug class on the index date. Broad use of the INSTIs class in the database started after 2010, 2 years after INSTIs were introduced in Japan in July 2008. Therefore, the main results reported in the present study are derived from patients who started an ART regimen between 2011 and 2019 (n = 16,069). Patient disposition is outlined in Supplemental Fig. 1.

### Distribution of anchor drug class and backbone drugs in ART regimens by year

**Figure 1:**
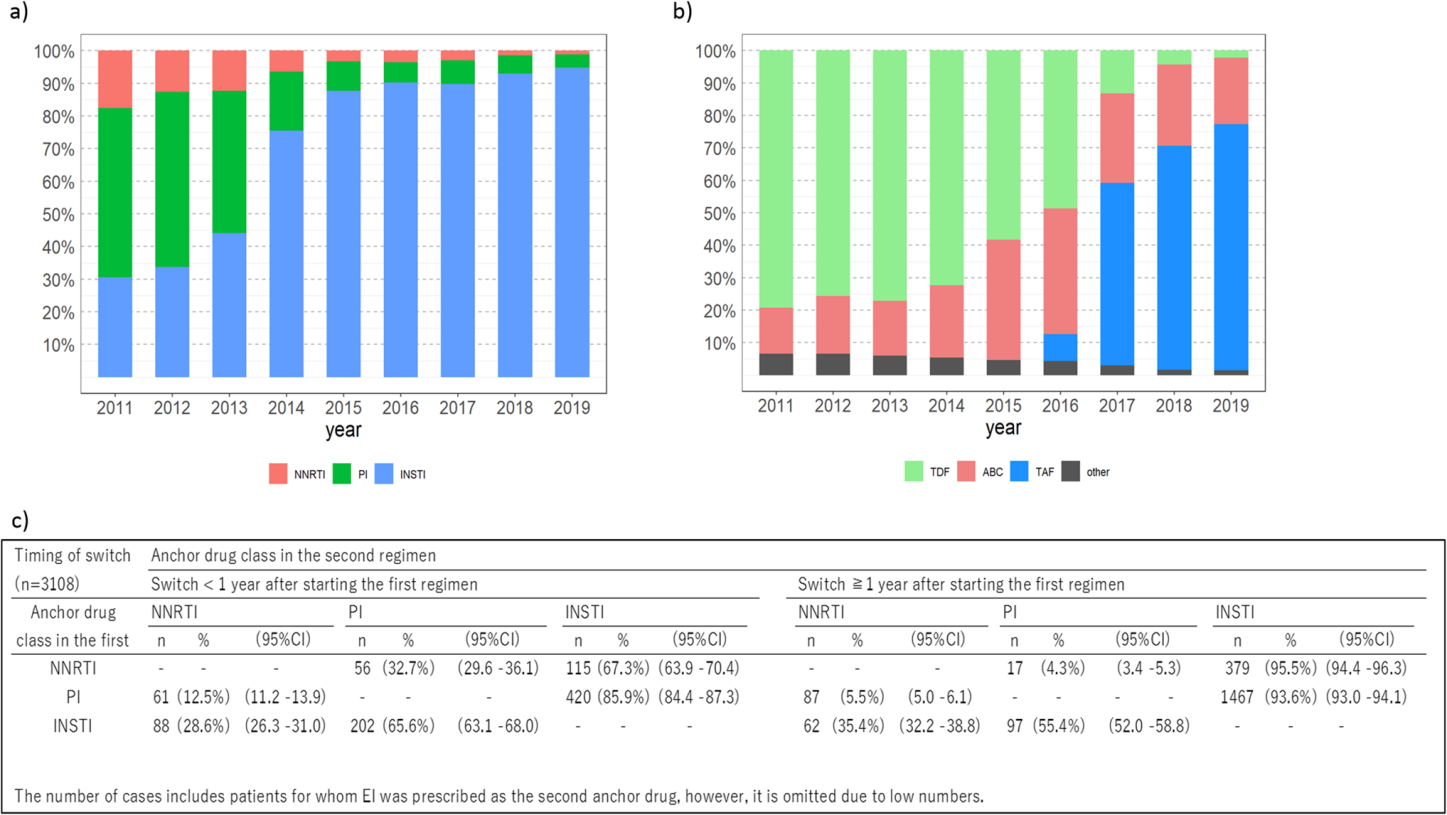
Distribution of (**a**) anchor and (**b**) backbone drug classes prescribed in first ART regimens by year (2011‒2019) (n = 16,069). (**c**) Timing and pattern of anchor drug class switching by duration after first ART regimen (n = 3108). Anchor drugs: *NNRTI* Non-nucleoside reverse transcriptase inhibitor, *PI* Protease inhibitor, *INSTI* integrase strand transfer inhibitor. Backbone drugs: *ABC* Abacavir, *TDF* Tenofovir disoproxil fumarate, *TAF* Tenofovir alafenamide, others include 3TC: lamivudine, FTC: emtricitabine. The years of launch of the anchor drugs by class were as follows: NNRTI: etravirine (launched in 2009) and rilpivirine (2012); PI: darunavir (2013); and INSTI: raltegravir (2008), dolutegravir (DTG) (2014), DTG/ABC/3TC (2015), and elvitegravir/cobi/TDF/FTC (2013). Patients prescribed an entry inhibitor in combination with others as a backbone were excluded.

Prescriptions for NNRTIs and PIs as anchor drug classes in overall ART regimens decreased after 2011 (NNRTIs: 18–1% from 2011 to 2019; PIs: 52–4% from 2011 to 2019) (Fig. 1a). Contrary to these trends, prescriptions for INSTIs increased rapidly since 2011 and accounted for the majority of anchor drug classes prescribed in 2019 (30% in 2011 and 95% in 2019). Among backbone drug types, changes were observed starting from 2016. TDF was the predominant backbone drug type between 2011 and 2016 (80–50%), followed by ABC (14% in 2011 to 38% in 2016). TAF was the predominant backbone drug type from 2017 to 2019 (55% in 2017 to 70% in 2019) (Fig. 1b).

Of 16,069 patients who started an ART regimen during 2011–2019, 7.5% (n = 1204) were on NNRTIs, 24.3% (n = 3901) on PIs, and 68.2% (n = 10,964) on INSTIs (Supplemental Table 2). Patients on INSTIs had higher proportions of psychiatric disorders compared to patients on NNRTIs and PIs (Supplementary Table 2).

### Characteristics of patients who switched anchor drug classes

Of all patients who started an ART regimen from 2011 to 2019, 19.3% (3108 patients) switched anchor drug class. For each anchor drug class, 47.1% switched from NNRTIs (568/1204), 52.7% from PIs (2057/3901), and 4.4% from INSTIs (483/10,964) (Table 1). Amon the 3108 patients who switched ART regimen, 13.3% (n = 431) had multiple (≥ 2) switches while the majority of patients (86.1%) had one ART regimen switch only (Table 1). Due to the small percentages of multiple anchor drug switches in the total study population (NNRTIs 4.3%; PIs 5.0%, INSTIs 1.7%), our subsequent analysis hereafter focused on first-to-second anchor drug switch.

**Table 1:** Distribution of number of anchor class switches from 2011 to 2019 (n = 16,069).

First anchor class	n	Number of Switch	n	
NNRTI	1204	None	636	(52.8%)
		1	516	(42.9%)
		≥ 2	52	(4.3%)
PI	3901	None	1844	(47.3%)
		1	1860	(47.7%)
		≥ 2	197	(5.0%)
INSTI	10,964	None	10,481	(95.6%)
		1	301	(2.7%)
		≥ 2	182	(1.7%)

*NNRTI* Non-nucleoside reverse transcriptase inhibitor, *PI* Protease inhibitor, *INSTI* Integrase strand transfer inhibitor.

No significant differences were found in patients’ characteristics between those treated with these three predominant anchor drug classes. AIDS-defining illnesses were present in 60.5% of patients treated initially with INSTIs, whereas the proportions were lower in those treated initially with NNRTIs or PIs (51.4% and 50.9%, respectively) (Table 2). The proportion of patients with vascular diseases was highest among patients treated initially with INSTIs (8.3%) compared to those treated initially with NNRTIs (4.4%) or PIs (3.5%). The proportion of patients with psychiatric disorders was highest among patients treated initially with INSTIs (27.3%), followed by those with PIs (16.4%) and NNRTIs (14.6%) (Table 2).

**Table 2:** Characteristics of patients who switched anchor drug class from the first ART regimen (n = 3108).

Characteristic	Overall			NNRTI			PI			INSTI		
	N = 3108			N = 568			N = 2057			N = 483		
**Age group (years)**												
< 20		22	(0.7%)		–	–	About	10	–		–	–
20–29		569	(18.3%)		105	(18.5%)		387	(18.8%)		77	(15.9%)
30–39		1111	(35.7%)		192	(33.8%)		760	(36.9%)		159	(32.9%)
40–49		854	(27.5%)		158	(27.8%)		560	(27.2%)		136	(28.2%)
50–59		351	(11.3%)		77	(13.6%)		213	(10.4%)		61	(12.6%)
60–69		163	(5.2%)		24	(4.2%)		101	(4.9%)		38	(7.9%)
≥ 70		38	(1.2%)	About	10	–	About	20	–	About	10	–
**Sex**												
Male		2855	(91.9%)		529	(93.1%)		1883	(91.5%)		443	(91.7%)
Female		253	(8.1%)		39	(6.9%)		174	(8.5%)		40	(8.3%)
AIDS-defining illness		1632	(52.5%)		292	(51.4%)		1048	(50.9%)		292	(60.5%)
Diabetes		739	(23.8%)		120	(21.1%)		490	(23.8%)		129	(26.7%)
Dyslipidemia		577	(18.6%)		87	(15.3%)		398	(19.3%)		92	(19.0%)
Hypertension		328	(10.6%)		65	(11.4%)		185	(9.0%)		78	(16.1%)
Bone disorder		78	(2.5%)		–	–		52	(2.5%)	About	10	–
Vascular diseases		138	(4.4%)		25	(4.4%)		73	(3.5%)		40	(8.3%)
Angina		74	(2.4%)		15	(2.6%)		36	(1.8%)		23	(4.8%)
Stroke		62	(2.0%)		–	–		39	(1.9%)	About	10	–
Myocardial infraction		–	–		–	–		–	–		–	–
Kidney disease		133	(4.3%)		22	(3.9%)		75	(3.6%)		36	(7.5%)
Urolithiasis		86	(2.8%)		16	(2.8%)		46	(2.2%)		24	(5.0%)
Chronic kidney disease		49	(1.6%)		–	–		29	(1.4%)	About	10	–
Cancers		268	(8.6%)		39	(6.9%)		150	(7.3%)		79	(16.4%)
AIDS-defining cancers		188	(6.0%)		27	(4.8%)		102	(5.0%)		59	(12.2%)
Non-AIDS-defining cancers		103	(3.3%)		17	(3.0%)		57	(2.8%)		29	(6.0%)
Psychiatric disorders		553	(17.8%)		83	(14.6%)		338	(16.4%)		132	(27.3%)
Mania and depression		356	(11.5%)		47	(8.3%)		219	(10.6%)		90	(18.6%)
Anxious		260	(8.4%)		42	(7.4%)		155	(7.5%)		63	(13.0%)
Psychosis		113	(3.6%)		13	(2.3%)		71	(3.5%)		29	(6.0%)
Insomnia		15	(0.5%)		–	–		–	–		–	–
Dementia		–	–		–	–		–	–		–	–
Hepatitis B infection		343	(11.0%)		60	(10.6%)		231	(11.2%)		52	(10.8%)
Hepatitis C infection		221	(7.1%)		36	(6.3%)		141	(6.9%)		44	(9.1%)
**Hospitalization**												
Hospitalized		1436	(46.2%)		220	(38.7%)		924	(44.9%)		292	(60.5%)
Never		1672	(53.8%)		348	(61.3%)		1133	(55.1%)		191	(39.5%)
**Year of ART initiation**												
2011		844	(27.2%)		196	(34.5%)		564	(27.4%)		84	(17.4%)
2012		837	(26.9%)		137	(24.1%)		637	(31.0%)		63	(13.0%)
2013		671	(21.6%)		120	(21.1%)		488	(23.7%)		63	(13.0%)
2014		351	(11.3%)		64	(11.3%)		202	(9.8%)		85	(17.6%)
2015		167	(5.4%)		22	(3.9%)		75	(3.6%)		70	(14.5%)
2016		110	(3.5%)		15	(2.6%)		43	(2.1%)		52	(10.8%)
2017		81	(2.6%)		–	–	About	20	–		43	(8.9%)
2018	About	40	–		–	–	About	10	–	About	20	–
2019		–	–		–	–		–	–		–	–

Excluded “none switcher from Table 1. Values are expressed as number (percentage) unless specified otherwise.

*ART* Antiretroviral therapy, *NNRTI* Non-nucleoside reverse transcriptase inhibitor, *PI* Protease inhibitor, *INSTI* Integrase strand transfer inhibitor.

### Switching patterns of anchor drug classes

Of the 3108 patients who switched anchor drug classes from their first to second ART regimens, most patients treated initially with NNRTIs and PIs switched to INSTIs (67.3% [95% CI: 63.9%‒70.4%] and 85.9% [84.4%‒87.3%], respectively) < 1 year after starting the first regimen; 65.6% [95% CI: 63.1%‒68.0%) of patients treated initially with INSTIs switched to PIs in the second regimen (Fig. 1c). Of the patients who switched their anchor drug class ≥ 1 year after starting their first regimen, most of those treated initially with NNRTIs and PIs switched to INSTIs (95.5%, [95% CI: 94.4%‒96.3%] and 93.6% [95% CI: 93.0%‒94.1%], respectively), whereas of those treated initially with INSTIs, 55.4% [95% CI: 52.0%‒58.8%] switched to PIs (Fig. 1c).

### Switching of anchor drug classes in the ART regimens

**Figure 2:**
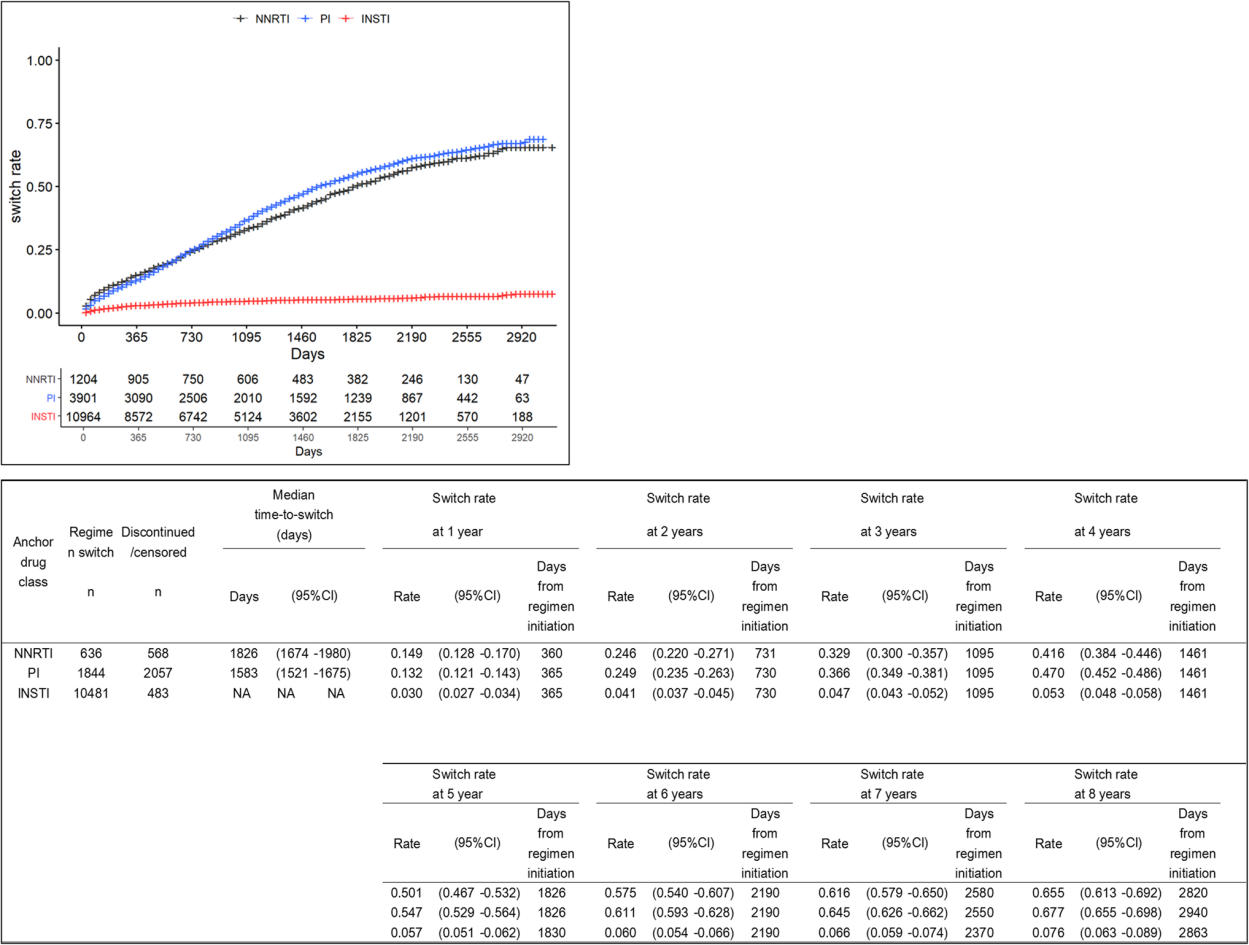
Time-to-switch of ART regimens according to anchor drug class from 2011 to 2019. The median time-to-switch and switch rates of anchor drug classes were estimated by Kaplan–Meier analysis. Brookmeyer–Crowley and Greenwood methods were used to estimate 95% confidence intervals for switch rates and median days, respectively. *ART* Antiretroviral therapy, *NNRTI* Non-nucleoside reverse transcriptase inhibitor, *PI* Protease inhibitor, *INSTI* Integrase strand transfer inhibitor, *EI* Entry inhibitor, *CI* Confidence interval. EI was excluded from the table as only one patient was prescribed EI regimen.

The switch rates for both NNRTIs and PIs increased constantly over 8 years (from 14.9% to 65.5% and 13.2% to 67.7%, respectively), whereas patients taking INSTIs maintained a low switch rate (from 3.0 to 7.6%) (Fig. 2). NNRTIs and PIs had median time-to-switch of 1826 and 1583 days, respectively, but INSTIs had a low switch rate of 7.6% at 8 years, so time-to-switch could not be obtained. Log-rank tests showed significant differences in switch rates of any pairs of anchor drug classes at years one through eight (all *p* < 0.05). Subgroup stratification by age of first ART prescription, sex, and status of AIDS diseases at first ART prescription showed no noticeable difference in switch pattern between the subgroups (Supplemental Fig. 2).

### Switching of anchor drug class-based backbone drugs in the ART regimens

**Figure 3:**
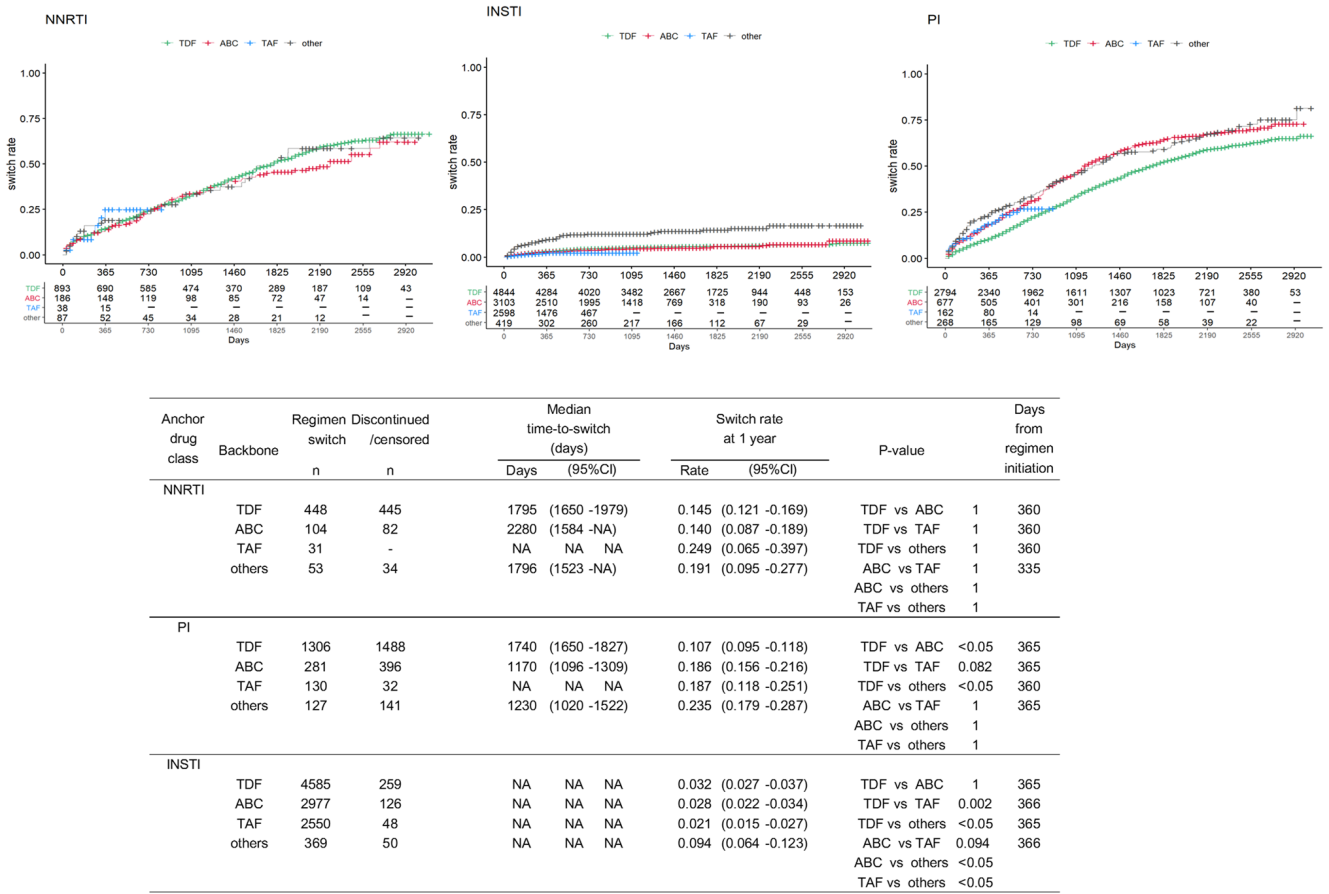
Time-to-switch of ART regimens by anchor drug class-based backbone type from 2011 to 2019. The median time-to-switch and switch rates of anchor drug classes were estimated by Kaplan–Meier analysis. Brookmeyer–Crowley and Greenwood methods were used to estimate 95% confidence intervals for switch rates and median days, respectively. Switch rates were compared between drug classes by using log-rank tests. The Bonferroni method was performed to adjust *p* values on multiple comparison. *ART* Antiretroviral therapy, *NNRTI* Non-nucleoside reverse transcriptase inhibitor, *PI* Protease inhibitor, *INSTI* Integrase strand transfer inhibitor, *ABC* Abacavir, *TDF* Tenofovir disoproxil fumarate, *CI* Confidence interval. Entry inhibitor (EI) was excluded from the table as only one patient was prescribed the EI regimen.

In patients receiving NNRTIs, the switch rates at 1 year varied between backbone drugs, with the lowest rate in ABC backbone drugs (14.0%). In patients receiving PIs, the lowest rate was for TDF backbone drugs (10.7%) (Fig. 3). In the PI group, significant differences were found in the switching rates between TDF and ABC and between TDF and other backbone drugs (all *p* < 0.05). In the INSTIs group, equally low switch rates were observed at 1 year for TAF (2.1%), ABC (2.8%), and TDF (3.2%). The switch rate for TAF was slightly lower than for TDF and ABC; the highest switch rate was using other backbone drugs (9.4%) (Fig. 3).

### Assessment of potential confounding factors associated with regimen switch

**Figure 4:**
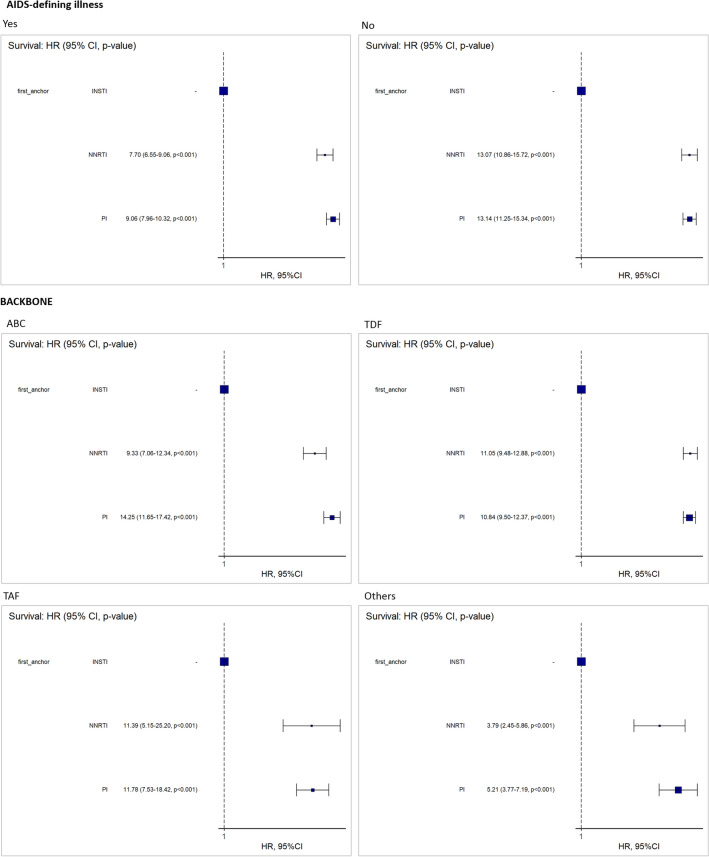
Hazard ratio for switching of each anchor drug class stratified by AIDS-defining illness and backbone drugs. *NNRTI* Non-nucleoside reverse transcriptase inhibitor, *PI* Protease inhibitor, *INSTI* Integrase strand transfer inhibitor, *TAF* Tenofovir alafenamide, *TDF* Tenofovir disoproxil fumarate, *ABC* Abacavir, *CI* Confidence interval. Variables, including the type of backbone, anchor drug class, AIDS-defining illness, and two interaction terms (between anchor drug class and AIDS-defining illness and between anchor drug class and type of backbone) were selected in the Cox regression analysis. Subsequently, the HR was calculated for anchor drug class stratified by each interaction term (AIDS-defining illness and type of backbone). The filled circles indicate the hazard ratio, and the horizontal lines indicate the 95% CI.

AIDS-defining illness, backbone type, and anchor drug class, and interaction terms between anchor drug class and AIDS-defining illness and between anchor drug class and type of backbone were selected for inclusion in the Cox regression analysis model. Subsequently, HRs were calculated for anchor drug class stratified by each interaction term with anchor drug class (AIDS-defining illness and backbone types) (Fig. 4). The HRs were consistently higher in regimens with PIs and NNRTIs compared with those with INSTIs, regardless of the presence of AIDS-defining illness (HRs 7.70‒9.06 for presence of AIDS, 13.07‒13.14 for non-AIDS) or backbone type (HRs 11.39‒11.78 for TAF, 10.84‒11.05 for TDF, and 9.33‒14.25 for ABC) (Fig. 4).

## Discussion

The present study is the first to use the cross-sectional, nationwide Japanese NDB to analyze treatment data of HIV-positive Japanese persons. Analyses of switch rates, time-to-switch, and switching patterns of anchor drug classes of ART regimens showed that the most common switching pattern of anchor drug class was from NNRTIs or PIs to INSTIs. Switch rates for NNRTIs and PIs increased continuously over 8 years, whereas initial INSTIs regimens maintained low switch rates, validating the results of our preliminary study^13^.

Among PLWH enrolled in the present study, INSTIs was the anchor drug class prescribed most frequently, and TAF agents (about two-thirds of backbone prescriptions) was the predominant initial backbone for the latest 3 years. The 2017 HIV treatment guidelines published in Japan^17^ reinforced these trends for initial ART regimens, showing that prescriptions for INSTIs agents [e.g., raltegravir (RAL) and dolutegravir (DTG)] as initial anchor drug classes increased between 2012 and 2016; TDF was prescribed most frequently as a backbone drug followed by ABC in the same timeframe. Despite INSTIs is equivalent to NNRTIs and PIs in terms of effectiveness, the increasing preference of INSTIs could be due to less side effects. NNRTIs may result in neuropsychiatric symptoms such as nightmares, and PI may result gastrointestinal symptoms. In addition, in recent years there are increasing number of new formulations containing INTIs being released. There were some concerns regarding switching of backbone drugs in the literature; PLWH who switched from a TDF-based to a TAF-based cART regimen showed increased low-density lipoprotein (LDL) values exceeding their cardiovascular risk targets^18,19^. In the present study, equally low switch rates were observed at 1 year for TAF, ABC, and TDF. However, the true impact of TAF on lipid profiles or cardiovascular risk was not evaluated and requires further study.

Over the 8-year study period, the switch rates of anchor drug classes NNRTIs and PIs increased steadily. Switching anchor drug class also increased 20% over 3 years in Europe and the United States in treatment-naïve PLWH treated initially with both NNRTIs and PIs^12^. The majority of patients in the present study who were treated initially with NNRTIs and PIs switched to INSTIs regardless of switching times and/or backbone drugs, with low switch rates thereafter. Consistent with findings reported by other investigators^20–22^ our results also showed that INSTIs may be the most durable anchor drug class for PLWH on ART regimens, regardless of backbone drugs in the first ART regimen. Analysis of a large dataset of HIV-positive patients confirmed that initial INSTI-based regimens combined with TDF, TAF, or ABC were all potent and well tolerated without significant virological failure; only a small percentage of patients (12%) discontinued INSTIs regimens, and DTG showed the lowest risk of virological failure^23^. The durability and efficacy of DTG were also reported in patients who switched to INSTI-based regimens, and subsequent switches were less likely than with RAL^22^. Switching regimens appears to be more stable in virologically suppressed HIV-1-infected patients who receive INSTI-based regimens initially^21,24^.

Other possible explanations suggested for increased switching include the expansion of HIV/AIDS treatment programs in middle-income or resource-limited areas^25^. In addition, HIV-infected patients are more likely to be younger, less educated, and to have detectable HIV-1 DNA when switching to a second-line cART regimen, which may predispose to worse outcomes. Comparison of outcomes of second-line cART regimens between 1996–1998 and 2008–2010 reported that failure rates decreased as time progressed and were independent of the cART regimen; risk of virologic failure of second-line cART was also lower in patients who had undetectable HIV-1 DNA at the time of switching^26^. Increases in ART drug resistance may also explain multiple switches in treatment^27^.

Of comorbidities, the prevalence of AIDS-defining illnesses was the highest in those treated initially with INSTIs, whereas dyslipidemia and diabetes mellitus prevalence was higher among patients receiving NNRTIs. Multivariate analysis, after adjusting for confounders, showed that the risk of switching anchor drug classes was lower in patients prescribed INSTIs, regardless of the presence of AIDS-defining illness or type of backbone prescribed, supporting long-term continuation of INSTIs prescribed for the first ART regimen. Of note, recent literatures have informed clinicians to remain aware of possible metabolic comorbidity despite inconclusive findings in the literature. Rebeiro et al.^28^, reported that initiating first cART regimens with INSTIs or PIs vs NNRTIs may confer greater risk of diabetes mellitus, likely mediated through weight gain, while Ursenbach et al.^29^, reported that INSTIs were not statistically associated with new-onset diabetes. The potential issue of metabolic comorbidity is not investigated in our current study; however, it is an important topic that warrant further investigation.

### Limitations

The present study has several limitations. Firstly, the study population was confined to Japan, and thus the results cannot be generalized to other populations. Secondly, patients with more comorbid chronic illnesses or more complications of HIV infection may have been hospitalized in HIV-specialized institutions or institutions offering advanced medical care, which may limit generalizing results to all PLWH throughout Japan. Lastly, data were from an administrative database and certain clinical data from individual patients (e.g., adverse events, treatment failure, poor adherence) were unavailable to accurately determine ART regimen changes or drug selection, in addition, one of the limitations of NDB is the lack of information on disease severity, and immune status such as viral load and CD4 count. A prospective long-term study taking into consideration of immune status and severity of comorbid diseases is needed to confirm the durability of INSTIs as initial ART regimen drugs.

## Conclusions

This is the first report of switch rates and time-to-switch of ART regimens using large nationwide database, NDB, which not only contains information on the largest number of HIV-positive patients in Japan, but also is representative of the entire population in Japan. The foremost switching strategies for first-to-second ART regimens were from NNRTIs or PIs to INSTIs, and there was no observable difference in switch pattern between sex, age and status of AIDS disease at first ART regimen. Incorporating INSTIs as the anchor drugs in initial ART regimen maintains low switch rates, suggesting that INSTIs may be the most durable anchor drug class for PLWH on ART regimens, regardless of AIDS-defining illnesses or backbone drug types prescribed.

## Supplementary Information

Supplementary Figure 1.

Supplementary Figure 2.

Supplementary Tables.

Supplementary Legends.

## Data Availability

The data that support the findings of this study were available from Ministry of Health, Labour and Welfare (MHLW) but restrictions apply to the availability of these data. Only researchers who have submitted a request to the MHLW and have been approved by the MHLW in accordance with the National Database Guidelines shall use the data. In general, the datasets generated during and/or analyzed during the current study are not publicly available. Data are however available from the authors upon reasonable request in compliance with the National Database Guidelines and with permission MHLW.

## Abbreviations

ABC: Abacavir
ART: Antiretroviral therapy
cART: Combination regimens
EIs: Entry inhibitors
HIV: Human immunodeficiency virus
INSTIs: Integrase strand transfer inhibitors
NDB: National Database of Health Insurance Claims and Specific Health Checkups of Japan
NNRTIs: Non-nucleoside reverse transcriptase inhibitors
PIs: Protease inhibitors
PLWH: People living with human immunodeficiency virus
TAF: Tenofovir alafenamide
TDF: Tenofovir disoproxil fumarate
