# Supplementary figures and images for: Analysis of antiretroviral therapy switch rate and switching pattern for people living with HIV from a national database in Japan
Source: Sci Rep. 2022 Feb 2;12:1732. doi: 10.1038/s41598-022-05816-5 (PMC8810755; doi:10.1038/s41598-022-05816-5)

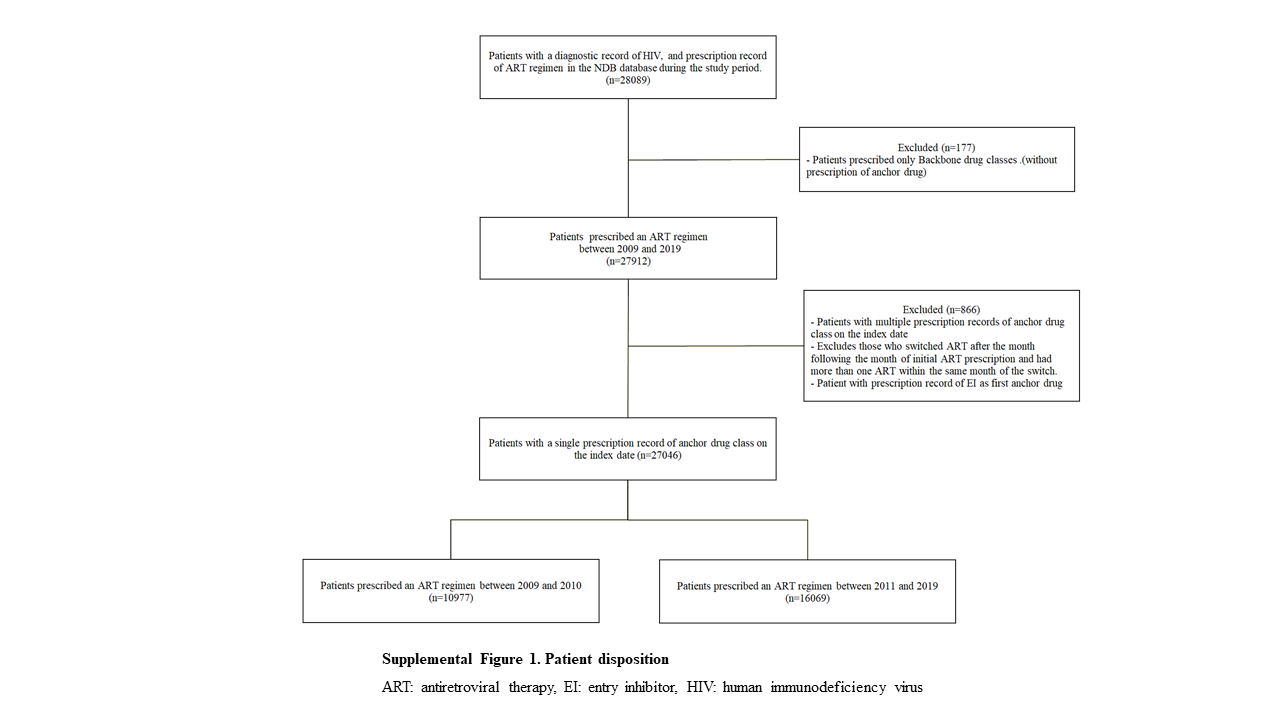

Supplement: Supplementary file 1 — Supplementary Figure 1. [file 41598_2022_5816_MOESM1_ESM.tif]

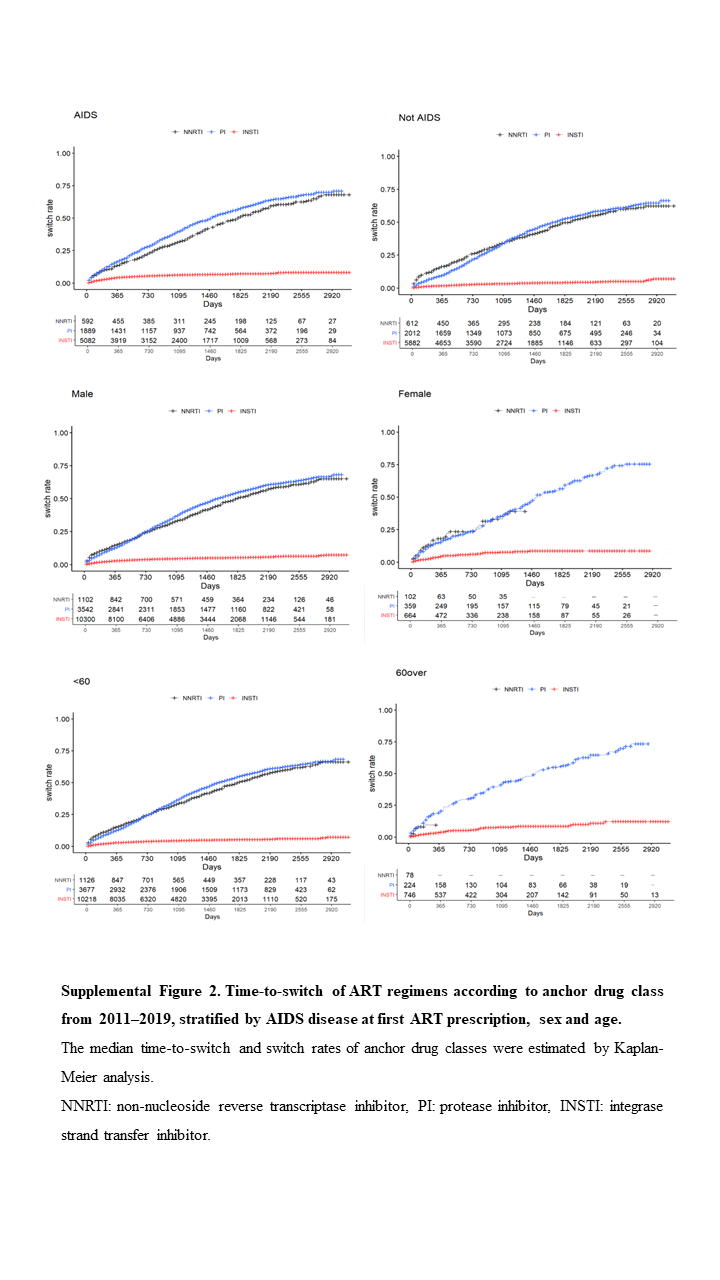

Supplement: Supplementary file 2 — Supplementary Figure 2. [file 41598_2022_5816_MOESM2_ESM.tif]
