# Supplementary material for: Analysis of antiretroviral therapy switch rate and switching pattern for people living with HIV from a national database in Japan
Source: Sci Rep. 2022 Feb 2;12:1732. doi: 10.1038/s41598-022-05816-5 (PMC8810755; doi:10.1038/s41598-022-05816-5)
Supplement: Supplementary file 3 — Supplementary Tables. [file 41598_2022_5816_MOESM3_ESM.docx]

**Supplement Table 1**. ICD-10 codes used to identify AIDS-defining illnesses and comorbidities

| Disease name | ICD-10 code |
| --- | --- |
| **AIDS-defining illnesses** |  |
| HIV non-tuberculous mycobacteria | B20.0 |
| HIV cytomegalovirus infection | B20.2 |
| HIV other viral infections | B20.3 |
| HIV candidiasis | B20.4 |
| HIV Pneumocystis carinii pneumonia | B20.6 |
| HIV Kaposi's sarcoma | B21.0 |
| HIV Burkitt's lymphoma | B21.1 |
| HIV non-Hodgkin's lymphoma | B21.2 |
| HIV encephalopathy | B22.0 |
| HIV lymphoid interstitial pneumonitis | B22.1 |
| Slim disease | B22.2 |
| HIV other specified conditions | B23.8 |
| AIDS | B24 |
| AIDS-related complex | B24 |
| Malignant neoplasm of the cervix uteri | C53 |
| **AIDS-defining cancers** |  |
| Kaposi sarcoma | B21.0, C46 |
| Burkitt lymphoma | B21.1, C83.7 |
| Non-Hodgkin lymphoma | B21.2, C82-C85 |
| Malignant neoplasm of the cervix uteri | C53 |
| **Non-AIDS-defining cancers** |  |
| Cancer other than the above | C00-97 （exclude C46, C53, C82-C85, C83.7） |
| **Comorbidities** |  |
| HIV-related diseases | ‒ |
| Cytomegaloviral disease | B25, B45, B59, C46.9, C81 |
| Hypertension | I10, I12, I14–15 |
| Dyslipidemia | ‒ |
| Hypercholesterolemia or hyperlipidemia | E78.0–78.5 |
| Hepatitis B infection | B18.1 |
| Hepatitis C infection | B18.2 |
| Diabetes | ‒ |
| Type 2 diabetes | E11‒14 |
| Bone disorder | ‒ |
| Osteoporosis | M80‒81 |
| Vascular disease | ‒ |
| Myocardial infarction | I21–22 |
| Stroke | I64 and related receipt diagnosis codes |
| Angina pectoris | I20 |
| Hypertensive heart and renal diseases | I11, I13 |
| Psychiatric disorders | ‒ |
| Mania and depression | F30–32 |
| Anxiety | F40–41 |
| Psychosis | F20–29 |
| Dementia | F01, F03 |
| Insomnia | F51 |
| Kidney disease | ‒ |
| Chronic kidney disease | N18–19 |
| Urolithiasis | N20–21 |
| Malignancies | B21.0-21.2, C00–97 |

ICD-10: International Statistical Classification of Diseases and Related Health Problems 10th Revision

**Supplement Table 2**. Patient demographics and characteristics according to anchor drug class from 2011‒2019 (n = 16,069)

|  |  |  | **Overall** |  |  |  | **NNRTI** |  |  |  | **PI** |  |  |  | **INSTI** |  |
| --- | --- | --- | --- | --- | --- | --- | --- | --- | --- | --- | --- | --- | --- | --- | --- | --- |
|  | **Characteristic** |  | **N = 16,069** | |  |  | **N = 1,204** | |  |  | **N = 3,901** | |  |  | **N = 10,964** | |
|  | Age group(years) |  |  |  |  |  |  |  |  |  |  |  |  |  |  |  |
|  | <20 |  | 83 | (0.5%) |  |  | - | - |  |  | 25 | (0.6%) |  | about | 50 | - |
|  | 20-29 |  | 3,198 | (19.9%) |  |  | 233 | (19.4%) |  |  | 723 | (18.5%) |  |  | 2,242 | (20.4%) |
|  | 30-39 |  | 5,447 | (33.9%) |  |  | 404 | (33.6%) |  |  | 1,457 | (37.3%) |  |  | 3,586 | (32.7%) |
|  | 40-49 |  | 4,498 | (28.0%) |  |  | 326 | (27.1%) |  |  | 1,047 | (26.8%) |  |  | 3,125 | (28.5%) |
|  | 50-59 |  | 1,795 | (11.2%) |  |  | 155 | (12.9%) |  |  | 425 | (10.9%) |  |  | 1,215 | (11.1%) |
|  | 60-69 |  | 847 | (5.3%) |  |  | 60 | (5.0%) |  |  | 190 | (4.9%) |  |  | 597 | (5.4%) |
|  | ≥70 |  | 201 | (1.3%) |  | about | 20 | - |  |  | 34 | (0.9%) |  | about | 150 | - |
|  | Sex |  |  |  |  |  |  |  |  |  |  |  |  |  |  |  |
|  | Male |  | 14,944 | (93.0%) |  |  | 1,102 | (91.5%) |  |  | 3,542 | (90.8%) |  |  | 10,300 | (93.9%) |
|  | Female |  | 1,125 | (7.0%) |  |  | 102 | (8.5%) |  |  | 359 | (9.2%) |  |  | 664 | (6.1%) |
|  | AIDS-defining illness |  | 7,563 | (47.1%) |  |  | 592 | (49.2%) |  |  | 1,889 | (48.4%) |  |  | 5,082 | (46.4%) |
|  | Diabetes |  | 3,702 | (23.0%) |  |  | 248 | (20.6%) |  |  | 888 | (22.8%) |  |  | 2,566 | (23.4%) |
|  | Dyslipidemia |  | 2,993 | (18.6%) |  |  | 189 | (15.7%) |  |  | 726 | (18.6%) |  |  | 2,078 | (19.0%) |
|  | Hypertension |  | 1,814 | (11.3%) |  |  | 123 | (10.2%) |  |  | 347 | (8.9%) |  |  | 1,344 | (12.3%) |
|  | Bone disorder |  | 466 | (2.9%) |  |  | 23 | (1.9%) |  |  | 99 | (2.5%) |  |  | 344 | (3.1%) |
|  | Vascular diseases |  | 833 | (5.2%) |  |  | 46 | (3.8%) |  |  | 149 | (3.8%) |  |  | 638 | (5.8%) |
|  | Angina |  | 445 | (2.8%) |  |  | 24 | (2.0%) |  |  | 78 | (2.0%) |  |  | 343 | (3.1%) |
|  | Stroke |  | 399 | (2.5%) |  |  | 17 | (1.4%) |  |  | 76 | (1.9%) |  |  | 306 | (2.8%) |
|  | Myocardial infraction |  | 70 | (0.4%) |  |  | - | - |  |  | - | - |  |  | 53 | (0.5%) |
|  | Kidney disease |  | 764 | (4.8%) |  |  | 40 | (3.3%) |  |  | 149 | (3.8%) |  |  | 575 | (5.2%) |
|  | Urolithiasis |  | 487 | (3.0%) |  |  | 23 | (1.9%) |  |  | 94 | (2.4%) |  |  | 370 | (3.4%) |
|  | Chronic kidney disease |  | 290 | (1.8%) |  |  | 17 | (1.4%) |  |  | 57 | (1.5%) |  |  | 216 | (2.0%) |
|  | Cancers |  | 1,517 | (9.4%) |  |  | 85 | (7.1%) |  |  | 313 | (8.0%) |  |  | 1,119 | (10.2%) |
|  | AIDS-defining cancers |  | 1,033 | (6.4%) |  |  | 51 | (4.2%) |  |  | 220 | (5.6%) |  |  | 762 | (7.0%) |
|  | Non-AIDS-defining cancers |  | 681 | (4.2%) |  |  | 40 | (3.3%) |  |  | 115 | (2.9%) |  |  | 526 | (4.8%) |
|  | Psychiatric disorders |  | 2,989 | (18.6%) |  |  | 141 | (11.7%) |  |  | 587 | (15.0%) |  |  | 2,261 | (20.6%) |
|  | Mania and Depression |  | 1,974 | (12.3%) |  |  | 81 | (6.7%) |  |  | 373 | (9.6%) |  |  | 1,520 | (13.9%) |
|  | Anxious |  | 1,424 | (8.9%) |  |  | 69 | (5.7%) |  |  | 276 | (7.1%) |  |  | 1,079 | (9.8%) |
|  | Psychosis |  | 705 | (4.4%) |  |  | 26 | (2.2%) |  |  | 125 | (3.2%) |  |  | 554 | (5.1%) |
|  | Insomnia |  | 75 | (0.5%) |  |  | - | - |  | about | 10 | - |  |  | 57 | (0.5%) |
|  | Dementia |  | 24 | (0.1%) |  |  | - | - |  |  | - | - |  | about | 10 | - |
|  | Hepatitis B infection |  | 1,740 | (10.8%) |  |  | 115 | (9.6%) |  |  | 414 | (10.6%) |  |  | 1,211 | (11.0%) |
|  | Hepatitis C infection |  | 1,233 | (7.7%) |  |  | 88 | (7.3%) |  |  | 288 | (7.4%) |  |  | 857 | (7.8%) |
|  | Hospitalization |  |  |  |  |  |  |  |  |  |  |  |  |  |  |  |
|  | Hospitalized |  | 7,553 | (47.0%) |  |  | 425 | (35.3%) |  |  | 1,697 | (43.5%) |  |  | 5,431 | (49.5%) |
|  | Never |  | 8,516 | (53.0%) |  |  | 779 | (64.7%) |  |  | 2,204 | (56.5%) |  |  | 5,533 | (50.5%) |
|  | Year of ART initiation |  |  |  |  |  |  |  |  |  |  |  |  |  |  |  |
|  | 2011 |  | 1,970 | (12.3%) |  |  | 347 | (28.8%) |  |  | 1,021 | (26.2%) |  |  | 602 | (5.5%) |
|  | 2012 |  | 1,983 | (12.3%) |  |  | 251 | (20.8%) |  |  | 1,064 | (27.3%) |  |  | 668 | (6.1%) |
|  | 2013 |  | 2,065 | (12.9%) |  |  | 254 | (21.1%) |  |  | 899 | (23.0%) |  |  | 912 | (8.3%) |
|  | 2014 |  | 2,017 | (12.6%) |  |  | 131 | (10.9%) |  |  | 364 | (9.3%) |  |  | 1,522 | (13.9%) |
|  | 2015 |  | 1,965 | (12.2%) |  |  | 64 | (5.3%) |  |  | 178 | (4.6%) |  |  | 1,723 | (15.7%) |
|  | 2016 |  | 1,930 | (12.0%) |  |  | 69 | (5.7%) |  |  | 119 | (3.1%) |  |  | 1,742 | (15.9%) |
|  | 2017 |  | 1,926 | (12.0%) |  |  | 56 | (4.7%) |  |  | 140 | (3.6%) |  |  | 1,730 | (15.8%) |
|  | 2018 |  | 1,716 | (10.7%) |  | about | 20 | - |  | about | 90 | - |  |  | 1,594 | (14.5%) |
|  | 2019 |  | 497 | (3.1%) |  |  | - | - |  | about | 20 | - |  |  | 471 | (4.3%) |

ART: antiretroviral therapy, NNRTI: non-nucleoside reverse transcriptase inhibitor, PI: protease inhibitor, INSTI: integrase strand transfer inhibitor

Values are expressed as number (percentage) unless specified otherwise.
