# Supplementary material for: Analysis of antiretroviral therapy switch rate and switching pattern for people living with HIV from a national database in Japan
Source: Sci Rep. 2022 Feb 2;12:1732. doi: 10.1038/s41598-022-05816-5 (PMC8810755; doi:10.1038/s41598-022-05816-5)
Supplement: Supplementary file 4 — Supplementary Legends. [file 41598_2022_5816_MOESM4_ESM.docx]

**Supplementary Figure 1. Patient Disposition**

ART: antiretroviral therapy, EI: entry inhibitor, HIV: human immunodeficiency virus

**Supplement Figure 2. Time-to-switch of ART regimens according to anchor drug class from 2011‒2019, stratified by AIDS disease at first ART prescription, sex and age.**

The median time-to-switch and switch rates of anchor drug classes were estimated by Kaplan-Meier analysis.

NNRTI: non-nucleoside reverse transcriptase inhibitor, PI: protease inhibitor, INSTI: integrase strand transfer inhibitor.
